# The Complete Chloroplast Genome of *Plukenetia volubilis* Provides Insights Into the Organelle Inheritance

**DOI:** 10.3389/fpls.2021.667060

**Published:** 2021-04-23

**Authors:** Simón Villanueva-Corrales, Camilo García-Botero, Froilán Garcés-Cardona, Viviana Ramírez-Ríos, Diego F. Villanueva-Mejía, Javier C. Álvarez

**Affiliations:** ^1^BEC Research Group, Biological Sciences Department, EAFIT University, Medellín, Colombia; ^2^CIBIOP Research Group, Biological Sciences Department, EAFIT University, Medellín, Colombia

**Keywords:** oilseed crop, *de novo* assembly, non-model plant, Sacha inchi, long reads ONT

## Abstract

*Plukenetia volubilis* L. (Malpighiales: Euphorbiaceae), also known as Sacha inchi, is considered a promising crop due to its high seed content of unsaturated fatty acids (UFAs), all of them highly valuable for food and cosmetic industries, but the genetic basis of oil biosynthesis of this non-model plant is still insufficient. Here, we sequenced the total DNA of Sacha inchi by using Illumina and Nanopore technologies and approached a *de novo* reconstruction of the whole nucleotide sequence and the organization of its 164,111 bp length of the chloroplast genome, displaying two copies of an inverted repeat sequence [inverted repeat A (IRA) and inverted repeat B (IRB)] of 28,209 bp, each one separating a small single copy (SSC) region of 17,860 bp and a large single copy (LSC) region of 89,833 bp. We detected two large inversions on the chloroplast genome that were not presented in the previously reported sequence and studied a promising cpDNA marker, useful in phylogenetic approaches. This chloroplast DNA (cpDNA) marker was used on a set of five distinct Colombian cultivars of *P. volubilis* from different geographical locations to reveal their phylogenetic relationships. Thus, we evaluated if it has enough resolution to genotype cultivars, intending to crossbreed parents and following marker’s trace down to the F1 generation. We finally elucidated, by using molecular and cytological methods on cut flower buds, that the inheritance mode of *P. volubilis* cpDNA is maternally transmitted and proposed that it occurs as long as it is physically excluded during pollen development. This *de novo* chloroplast genome will provide a valuable resource for studying this promising crop, allowing the determination of the organellar inheritance mechanism of some critical phenotypic traits and enabling the use of genetic engineering in breeding programs to develop new varieties.

## Introduction

*Plukenetia volubilis* L., also known as Sacha inchi, is a climbing, perennial, semi-woody, twining oilseed plant of the Euphorbiaceae family, endemic to tropical Peruvian Amazonia that grows mainly in tropical forests at altitudes between 200 and 1500 meters above sea level (MASL) (Gillespie, 1993; Krivankova et al., 2013). It is known that Sacha inchi has a rapid growth ratio, an easy adaptation to growth in nutrient-poor soils, short production cycles, high nutritional content, and displays a potential capacity to become an essential dietary source (Hamaker et al., 1992; Chirinos et al., 2013). For instance, its amino-acid profile presents higher fractions of tryptophan, cysteine, and tyrosine than other oil-seeds sources, reaching the Food and Agriculture Organization’s (FAO) highest standards for 2–5 years’ children dietary supplements, except for leucine and lysine (Hamaker et al., 1992). Also, it has been shown that the crop relevance of *P. volubilis* indeed relies on its high seed unsaturated fatty acid (UFA) content, particularly of α-linolenic acid or ω3 (12.8–16.0 g/100 g seed) and linoleic acid or ω6 (12.4–14.1 g/100 g seed) (Chirinos et al., 2016).

Likewise, it has been reported that the establishment of commercial plantations of this species generates many positive environmental impacts because it can be installed on degraded soils (Bordignon et al., 2012). Thanks to all of the above, the Sacha inchi industry has experienced a great demand in tropical countries, among others, Colombia, Peru, Ecuador, and Brazil, where the cultivation of this species has been increasing (Valente et al., 2017), without developed varieties and ecotypes with genetic stability. In those countries, the ecotypes have been rudimentary and are too difficult to track because of the lack of studies that have been made to understand its genetic diversity (Ocelák et al., 2015). This lack of studies makes it necessary to develop molecular mechanisms that characterize the species, recognize ecotypes, and generate successful seed marketing; responses can be faced by knowing the Sacha inchi chloroplast genome. Insights obtained from the complete chloroplast genome sequence could enhance knowledge of plant biology and diversity for this species.

Chloroplast genomes have assembled notable contributions in diverse plant families, settling evolutionary relationships within phylogenetic clades. Moreover, chloroplast genome sequences have exposed considerable variation between plant species in terms of both sequence and structural variation (Daniell et al., 2016). This information advocates understanding the climatic adaptation of economically important crops, facilitating the breeding of closely related species, developing propagation technologies, genetic engineering applications, and identifying and conserving valuable traits (Wambugu et al., 2015). In addition to improving our understanding of plant biology and evolution, chloroplast genomics research has critical translational applications, such as conferring protection against biotic and abiotic stress and the development of vaccines and biopharmaceuticals in edible crops plants (Brozynska et al., 2016). All of them are significant aspects to be generated in Sacha inchi. Contributing to baseline, Hu et al. (2018) reported the first *P. volubilis* chloroplast genome sequence (161,733 bp, still unverified GenBank accession number: MF062253) from Xishuangbanna Tropical Botanical Garden in China. However, *P. volubilis* chloroplast’s genetics have not been deeply studied.

The chloroplast genome usually occurs in multiple copies within the organelle. It consists of long circular or linear DNA molecules, generally ranging from 120 to 180 kb in angiosperms and 160 to 164 kb in the Euphorbiaceae family (Rivarola et al., 2011; Tangphatsornruang et al., 2011; Zhang et al., 2019). It has a quadripartite structure characterized by two copies of a large inverted repeat A (IRA) and an inverted repeat B (IRB), separating the small single copy (SSC) and the large single copy (LSC) regions. Changes in chloroplast genomes’ composition and structure, such as gene losses and rearrangements, have been documented for *Passiflora edulis, Cistanche deserticola, Hevea brasiliensis* (Tangphatsornruang et al., 2011; Li et al., 2013; Cauz-Santos et al., 2017).

Based on CBOL (The Consortium for the Barcode of Life) evidence, four coding sequences (*matK*, *rbcL*, *rpoB*, and *rpoC1*) and three non-coding nucleotide inter-genic spacers (ISs) (*atpF-atpH*, *psbA-trnH^GUG^*, and *psbK-psbI*) from the chloroplast were suggested to be the adequate plant barcodes for phylogenetic relationships (CBOL Plant Working Group, 2009). Although DNA barcode has been adopted for decades as an investigation system for interspecific taxonomic discrimination, recent evidence suggests that this method may also be applied to plant intraspecific identification and population studies (Ünsal et al., 2019). Among these, *psbA-trnH^GUG^* was used to establish the evolutive relationships in tribe *Plukenetieae*, which used 153 accessions covering 93 species (Cardinal-McTeague and Gillespie, 2016).

In the Euphorbiaceae family and *P. volubilis*, chloroplast genes such as *rbcL*, *matK*, *ndhF*, and *trnL-F* have been used to study evolutionary relationships at higher taxonomic levels and distribution (Wurdack et al., 2005; Cardinal-McTeague et al., 2019). Nowadays, it is possible to generate entire chloroplast genomes and analyze entire chloroplast gene sequences to determine high-resolved phylogenies (Menezes et al., 2018). Third-generation sequencing technologies producing longer DNA reads have begun to produce high-quality assemblies for complex plant genomes (Li et al., 2018). Oxford Nanopore Technology (ONT) sequencing allows generating an entire chloroplast genome assembled into a single large contig, with a high degree of accuracy and much greater coverage due to longer read lengths (Belser et al., 2018).

Chloroplast DNA (cpDNA) barcodes are also helpful to prove the organelle’s inheritance combined with cytological approaches. In all plant taxa, current evidence shows three possible ways by which organellar DNA (oDNA) could be inherited: maternally, paternally, or biparentally (Reboud and Zeyl, 1994). Angiosperms seem to display mainly a maternal inheritance mode of its chloroplasts (Corriveau and Coleman, 1988; Greiner et al., 2015). However, recent studies have shown that some of them are indeed showing a potentially biparental plastid inheritance (PBPI) mode (i.e., the tendency of inheriting organelle genomes from both parents) (Zhang and Sodmergen, 2010).

In the present study, we approached a *de novo* reconstruction of the whole nucleotide sequence and the chloroplast genome organization using short and long reads technologies. We were able to localize genes, introns, and intergenic spacers and compare the structure of the cpDNA of *P. volubilis* from a Colombian cultivar with the cpDNA that has been reported in China. We found a useful cpDNA marker derived from a phylogenetic approach and used it on a set of five distinct cultivars of *P. volubilis* from different Colombian geographical locations. Thus, we evaluated if it has enough resolution to genotyping cultivars, intending to crossbreed parents and follow this marker in the F1 generation. We finally elucidated the inheritance mode of *P. volubilis* oDNA using both molecular and cytological methods on cut flower buds.

## Materials and Methods

### Plant Material

This research was performed at the Plant Biotechnology and Molecular Biology Laboratories of the Department of Biological Sciences at EAFIT University, located in Medellin, Colombia. Both leaves and seeds were collected from five *P. volubilis* cultivars from farms located in Antioquia (Colombia), showing different environmental conditions and ranging in an altitudinal gradient from 685 to 1501 MASL, having permission for this gathering issued by the National Authority for Environmental Licenses (ANLA), covered in resolution 1516 of 2014 (modified through resolution 1312 of 2015). Those samples were selected by their agronomic trials (Supplementary Figure 1) from individuals at each cultivar. Then, samples were entirely washed with distilled water, wiped and packed in bags to avoid light degradation, and stored at −20°C. Additionally, flower buds from Santa Rosa’s Cultivar 1 (named C1) were sampled in their first four developmental stages according to Cachique (2006) for cytological observations because it was regarded as promising cultivars after being assessed as suitable to be grown in dry agroclimatic areas. Sacha inchi cultivars C1–C5 are all deposited in the plant collection of the EAFIT’s University Plant Biotechnology Lab and are listed in Supplementary Table 1.

### DNA Extraction

To evaluate the cpDNA *psbA-trnH^GUG^* IS resolution and untangle the intraspecific variation in *P. volubilis* among Colombian cultivars, total DNA was extracted from leaf tissues of five plant cultivars across Antioquia, Colombia. This was made by displaying ecological and altitudinal variation (C1–C5, Supplementary Table 1) using the CTAB method (Doyle and Doyle, 1990), with the addition of a washing step with pure chloroform and the total elimination of isoamyl alcohol; additionally, the centrifugation times in each step were further reduced. The integrity of DNA was determined through 1% agarose gel electrophoresis, and the concentration was determined using Nanodrop 2000 (Thermo Scientific, Inc., CA, United States).

For ONT and Illumina sequencing, high molecular weight genomic DNA from the C1 leaves was isolated following Ramírez-Ríos et al. (2016) protocol, with some adaptations for plant DNA, as described in Supplementary Methods 1. Gel electrophoresis was used to evaluate the extract quality by size estimation, spectrophotometry (A260/A280 and A260/A230 ratios) was used for purity estimation, and Qubit 3 fluorometer (Invitrogen, Carlsbad, CA, United States) for total DNA extracted. DNA samples with an A260/A280 ratio close to 2 and an A260/A230 ratio above 1.5 were kept.

### Next-Generation Sequencing and Genome Assembly

Total DNA was sequenced using one lane of Illumina Hi-Seq 4000 paired-end per 150 nt and two flow cells of GridION (Oxford Nanopore Technologies) at the High-Throughput Sequencing Facility of the University of North Carolina at Chapel Hill, United States. Raw whole-genome sequencing paired-end reads were *de novo* pre-assembled using Norgal (Al-Nakeeb et al., 2017) with default settings. The resulting scaffolds were filtered based on the Norgal blast report: the best hit reference was a chloroplast; minimum scaffold length of 1000 bp; minimum identity 95%; and minimum alignment length of 200 bp. The filtered scaffold was used as seed for NOVOPlasty (v2.7.2) (Dierckxsens et al., 2016), which was used to assemble the same raw paired-end reads, set with a 100–200 kb genome range, a kmer size of 39, an insert size of 370, and the remaining parameters by default. The quality of the resulting sequence was further assessed using Pilon (v1.23) (Walker et al., 2014) as follows: raw paired-end reads were trimmed using Trimommatic-PE (v0.39) (Bolger et al., 2014) with a sliding window of width 4 and quality 25 and a min length of 50. All surviving (paired and unpaired) reads were mapped using BWA-MEM (v0.7.17) (Li et al., 2013). SAMtools (v1.9) (Li et al., 2009) were used to filter the unmapped reads and sort the resulting alignments. These were provided to Pilon, along with the generated sequence, with options –fix all and min depth of 60 and the remaining parameters as default.

Similarly, the structural quality of the assembly was assessed with ONT reads. Nanopore raw reads were basecalled with Guppy (v2.3.5+53a111f, Oxford Nanopore Technologies), using the flip-flop model for DNA, yielding a total of 4.699.073 reads with an N50 of 2 kbp. These reads were mapped with minimap2 (v2.17-r941) (Li, 2018) and visualized with Tablet (v1.17.08.17) (Milne et al., 2013).

No changes were suggested by Pilon, which indicates that every base in the assembled sequence is following the bases on the reads. On the other hand, misassemblies can be detected by inspecting the coverage profile of independent data on the assembly, as there would be drops in coverage in misassembled regions. A total of 956,508 mapped ONT reads generated a continuous smooth coverage of an average 3917x depth, where the LSC, SSC, and IRs could be visualized (Supplementary Figure 2), indicating the structural correctness of the assembly. The assembled chloroplast genome was hereafter called SI_cpDNA_C1 (C1).

### Chloroplast Genome Annotation and Comparison

The chloroplast sequence (SI_cpDNA_C1) was uploaded as a FASTA file to the Chlorobox portal and annotated using GeSeq (Tillich et al., 2017) tool using the following parameters: Circular and plastid sequence; annotate plastid IR, 85% protein search identity, and 85% rRNA, tRNA, and DNA search identity as BLAT search options; ARAGORN (v1.2.38) (Laslett and Canback, 2004) was selected as third-party tRNA annotator, with bacterial/plant chloroplast as genetic code, max intron length set to 2500 bp, and fixing introns. Since the automated annotation is error-prone, CDS were reviewed and manually curated, checking for correct start codons, lengths, and stop codons, and then the complete annotation was deposited under the GenBank accession number MW591569. SI_cpDNA_C1 was compared with MF062253.1 accession looking for nucleotide variants. Genome alignment was generated in MUMmer 3 (Kurtz et al., 2004) using the suite NUCmer, then the alignment output was filtered with delta-filter, and finally, single nucleotide polymorphisms (SNPs) were calculated with Dnadiff using default parameters. Scripts in Python and Biopython to filter and resume the data were documented in Jupyter notebooks and deposited in the Github repository for this work. All these processes and parameters are clarified in the repository associated with this article (Villanueva et al., 2020). To detect structural variants, SI_cpDNA_C1 was aligned to NC_016736 using NUCmer. The resulting alignments were used as input to MUMmerplot. Furthermore, SimpleSinteny (Veltri et al., 2016) was made using the 60 longest genes present in both chloroplasts.

### CpDNA *psbA-trnH^GUG^* Marker Amplification and Sequencing

To amplify the cpDNA *psbA-trnH^GUG^* IS fragment from each cultivar of *P. volubilis*, *trnH*^GUG^ (5’-CGCGCATGGTGGATTCACAATCC-3’) (Tate and Simpson, 2003) and *psbA* (3’-GTTATGCATGAACGTAATGCTC-5’) (Sang et al., 1997) primers were used (Supplementary Methods). Amplified PCR products from *psbA-trnH^GUG^* IS (∼500 bp) were sequenced using the Sanger method (Macrogen Inc., South Korea). Eventually, sequences were trimmed for base scores below 18 Phred score in a 5 base window average using Biopython libraries (Cock et al., 2009). Then, each *trnH*^GUG^ was locally aligned with the reverse complement of its *psbA* cultivar sequence. Each cultivar consensus region was reconstructed using the Smith and Waterman (1981) algorithm, with an identity more significant than 95%. Therefore, the species fragments’ identity was verified running the BLAST algorithm in the NCBI database, and the sequences were deposited in the GenBank database (Supplementary Table 1). Subsequently, these sequences were aligned using ClustalW (Thompson et al., 2003) on Geneious Pro ver. 11.1.5 and manually scanned the polymorphic regions.

### Phylogenetic Analyses Methods

Two main phylogenetic hypotheses were inferred using maximum likelihood (ML) and Bayesian inference (BI). Analysis of the aligned matrix with JmodelTest software ver. 2.1.10 (Darriba et al., 2012) showed that the TVM+I+G4 (Motoo, 1981) substitution model was the best model based on the delta Akaike information criterion (ΔAICc) and delta of Bayesian information criterion (ΔBIC) information criteria. Bayesian information criterion (BIC) for the same matrix showed the F81+G4 substitution model as the best choice. However, topologies for both models did not change (see Supplementary Table 4). RAxML-Ng ver. 0.9 (Kozlov et al., 2019) was used to reconstruct the best ML topology using five independent replicates; 1,000 bootstrap replicates were targeted to the best topology using DendroPy ver. 4.0 (Sukumaran and Holder, 2010) in a non-parametric bootstrapping fashion and consensus topology was visualized using FigTree ver. 1.4.3.

For BI, the aligned matrix file was edited using Beauti ver. 2.0 and updated the substitution model to TPM1uf + I and the strict molecular clock. Then, to infer the relationships, 1 × 10^7^ generations were run using Markov chain Monte Carlo (MCMC) algorithm, sampling one tree every 1,000 generations in BEAST ver. 2.5. The adequate sample size (ESS) values for all parameters were > 200, and they reached convergence and stationarity as determined by Tracer ver. 1.7.1 (Rambaut et al., 2014). Finally, the maximum credibility tree was generated using TreeAnnotator ver. 2.5 implementing a 10% burn-in and visualized using FigTree ver. 1.4.3. Both topologies (ML and BI) were summarized and edited using Inkscape ver. 0.9. Branch lengths from BI phylogeny were conserved, but both ML bootstrap (MLB) and Bayesian posterior probability (BPP) support values were depicted in the final tree.

### Inheritance Determination Based on Cytogenetic Analysis

Seeds of three cultivars (C1, C3, and C5) of *P. volubilis* were brought into cultivation in a greenhouse at EAFIT University, located in Medellin-Colombia. After germination, seedlings were placed in 11 cm pots within a mixture of 50% sand and 50% potting soil. Afterward, those plants were placed under controlled conditions (25°C, 2 months at 12 h light/12 h dark) before pollinations. Due to *P. volubilis* is an allogamous species with a high percentage of self-pollination (Cachique, 2006), controlled hand pollinations were undertaken (between November 13 and December 13, 2018) with C1, 3, and 5 (C1, C3, and C5, respectively). Before the cross-pollinations, all open flowers were removed from the inflorescence. The unopened buds were emasculated, and when the stigma became receptive (usually within two days), pollen was applied directly from the anthers of the pollen parent, following the Soda Straw Method (TNAU, 2015). The stigma of C5 was pollinated with pollen from C1 and C3. Reciprocal pollinations were also undertaken with C5 as male and C1 as female (Table 1). Once the first generation was generated, five seeds were collected and placed in 11-cm pots in a mixture of 50% sand and 50% potting soil. Afterward, the plants were placed under controlled conditions (25°C, 2 months at 12 h light/12 h dark). The leaves from the hybrids were collected and disposed of for DNA extraction and subsequent sequencing of the chloroplast’s intergenic *psbA-trnH^GUG^* region. Likewise, to visualize cpDNA, most developed flower buds (i.e., stage IV, just before anthesis, Cachique, 2006) from different individuals from C1 were immersed in a beaker with 5% sucrose overnight and later dried with towel paper. Then, water excess was absorbed by laying out the flower buds into silica gel inside a desiccation chamber for 30 min. Later, a Zeiss Stemi DV4 stereomicroscope was used to observe, and to tape dehiscent flower buds anthers over well depression slides to release the pollen. Afterward, two drops of germination media (GM) [sucrose 10%, boric acid (100 mg/L), magnesium sulfate (200 mg/L), nitrate (100 mg/L)] (modified from Brewbaker and Kwack, 1963) were spilled into the wells. Well depression slides were stored in a dark chamber at 25 ± 3°C for 24 h.

**TABLE 1:** Alignments of *psbA-trnH^GUG^* Inter-Genic Spacer sequences (nucleotides 321–345) from Colombian cultivars of Sacha inchi.

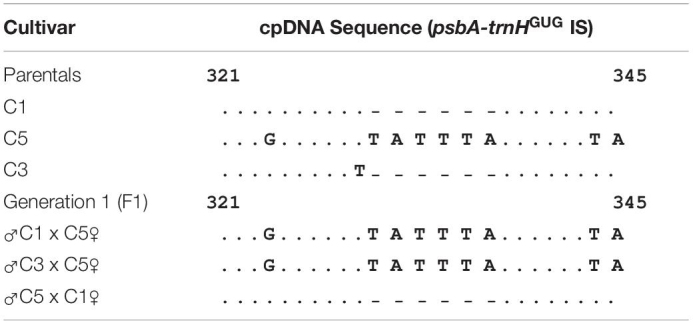

*Parentals; Santa Rosa cultivar (C1), Yarumal cultivar (C3), and San Luis cultivar (C5). Cross-pollinations were carried out between parentals (see section “Materials and Methods”), and F1 generation was genotyped. Dots mean similar nucleotides in the position, lines mean deletions, and letters are nucleotides with polymorphisms.*

Once the plants developed and opened their flowers, the pollen was collected to slide preparation for DAPI (NucBlue^TM^ Fixed Cell ReadyProbes^TM^ Reagent, ThermoFisher Scientific, United States) staining following three approaches: (i) pollen grains samples were left dried at room temperature before staining in the GM, (ii) fixation process in which pollen grains samples were treated sequentially with 10 and 30% ethanol and then incubated at 36°C to speed up evaporation, and (iii) slide preparation flower buds in four developmental stages were dehydrated by rinsing with glutaraldehyde and successive 20, 40, 60, 70, and 96% ethanol solutions (20 min each) and then dried in a BD23 Binder incubator at 37°C. Subsequently, histological sections of flower buds were carried out, embedding them in paraffin (Paraplast, 39601006, Leica) and cut at 10 μm with a microtome (RM2125 RTS, Leica). Flower buds histological slides were dehydrated at 65°C in a BD 23 Binder incubator for 1 h and then immersed in a succession of ethanol concentrations (60, 40, and 20%, 3 min each) for rehydration. Finally, slides were dehydrated with a succession of alcohols (20, 40, and 60%, 3 min each), rinsed with one drop of DAPI solution, and dried for 1 h. Images of pollen grains and histological sections were first processed using ZEN software from AxioCam Carl Zeiss and finally with FIJI® (2012).

## Results and Discussion

### Reconstruction of the Whole Chloroplast Genome of *P. volubilis* Using Next-Generation Sequencing Technologies

The hybrid strategy of ONT combined with Illumina was attempted for the first time in bacteria (Laver et al., 2015). Currently, it is a conventional hybrid method for relatively short genomes, such as cpDNA (Kang et al., 2019). This strategy of using a combination linking long and short reads may be the best approach to assembling chloroplast genomes due to its capacity to combine the benefits of the length of long reads and the accuracy of short reads (Wang et al., 2018). Previously, the chloroplast genome sequence and assembly in *Oryza coarctata* (Wang et al., 2018) and *Eucalyptus pauciflora* (Mondal et al., 2018) were reported by using ONT and Illumina. In this research, these NGS technologies were combined to reconstruct a whole chloroplast genome; first, with the short reads technology, the Illumina Hiseq 4000 system gave a total of 56.711.152 pairs of reads (according to post chloroplast assembly mapping and counting with BWA-MEM). Second, 4.699.073 reads resulted from the ONT after subsequent base-calling, with an N50 of 2 kbp. After the assembly, the average depth of coverage of the Illumina reads on the consensus sequence was 40,560x, and the average GC content was 35.8%.

### Organization and Comparative Analysis of Chloroplast Genome of *P. volubilis*

**FIGURE 1:**
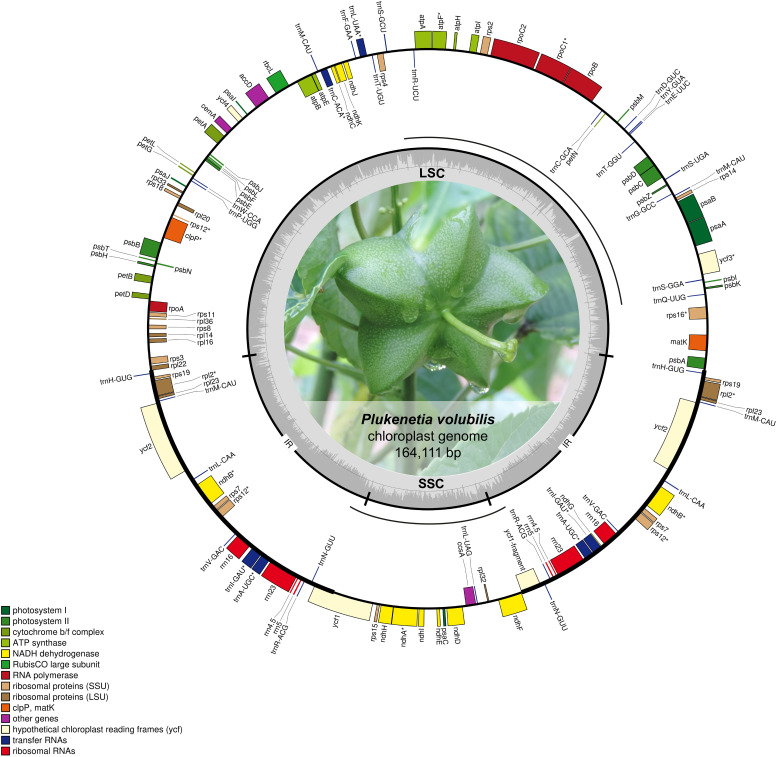
*P. volubilis* chloroplast genome map. Genes are represented as boxes inside or outside the large circle to indicate clockwise (inside) or counterclockwise (outside) transcription. The color of the gene boxes indicates the functional group to which the gene belongs. IR regions are in a smaller circle, and the inner has the GC content across the genome. LSC, large single-copy region; SSC, small single-copy region. Intron-containing genes are marked with an asterisk (“*”). Inversion regions are represented as discontinuous lines between the two circles.

The complete chloroplast genome of *P. volubilis* showed a single-circular molecule (Figure 1) similar to most other higher plants (Sato et al., 1999). The *P. volubilis* cpDNA resulted in a 164,111 bp length, 2378 bp more than Hu et al. (2018; Supplementary Table 2), displaying two copies of an inverted repeat (IRA and IRB) of 28,209 bp, each separating an SSC region of 17,860 bp and an LSC region of 89,833 bp (Figure 1). During gene annotation, the use of the accession NC_016736.1 of Ricinus *communis* as a reference, whose genome is the closest to *P. volubilis*, revealed unexpected asymmetries in the orientation of some rRNA genes on the IRs, in contrast to Arabidopsis *thaliana* (Sato et al., 1999), Nicotiana *tabacum* (Shinozaki et al., 1986), Glycine *max* (Saski et al., 2005), and others. Sequence level comparisons of these genes showed misannotations of the strand present in the *R. communis* chloroplast genome accession (see rRNA_orientation.ipynb in Villanueva et al., 2020). After reannotation without reference, the rRNA genes were found in the expected orientation. Annotation using Aragorn identified 131 genes (87 single-copy); 36 tRNAs identified eight rRNAs (four in each IR), 20 introns (in 18 genes), and 86 protein-coding genes, and an additional one fragmented (Supplementary Table 3).

From the tRNAs found, the normally *trnK-UUU*, which surrounds the *matK* gene, stands out as it presents an insertion that disrupts its anticodon converting it to *trnStop*-UUA. It is unclear if this tRNA is functional. However, as there is no other *trn*K, its malfunction would imply either a shortage or an alternative mechanism for lysine supply to the translation process. Regarding other genes, *atpF* has a conserved intron of 718 bp, *ycf1* has a fragmented copy of 1916 bp in IRB and *ycf3*. Also, *clpP* has two introns, and *rps*12 presents trans-splicing as it was also seen in Castor bean and found in bacterial and organellar genomes and thought to be ancestors of spliceosomal introns and retrotransposons in eukaryotes (Bonen, 2008; Lambowitz and Zimmerly, 2011).

We also reannotated the MF062253.1 genome due to mistakes assumed in the assembly with the reference *R. communis*, and the annotation pipeline followed Mummer (Kurtz et al., 2004) and Autograph (Derrien et al., 2007) comparisons between Chinese genome and Colombian C1 genome showed two inversions in the LSC and SSC regions (Figure 1). Read coverage in this region was highly supported, and ONT reads were mapped confirming these inversions (Supplementary Figure 2). No duplications were found in the genomes, but seven GAPs in the Colombian genome summing 1782 bp were inserted, and six GAPs were deleted summing 596 bp. The variant analysis found 52 SNPs flanked by 20 exact base pair nucleotides match on both sides. Transition A: T→G: C and transversion A: T→C: G were the most abundant into the genomes with 32.7% of occurrence each. The genes with more SNPs relative to the Chinese accession were *rpoC1*, followed by the *ycf3* gene. We aligned the sequences of nucleotides and amino acids for both *rpoC1* and found several SNPs changing the protein’s coding sequence. We also compared our *rpoC1* with other *Euphorbiaceae*. We found that *R. communis* presented an indel of 26 aa (FSFARPIAKKPTFLRLRGSFEYEIQS) in the amino acid 145 N-terminal of the protein. Curiously, amino acid alignment of *rpoC1* with *Jatropha curcas* had a higher identity than *R. communis*, and there was no indel detected (see Snps_analysis.ipynb in Villanueva et al., 2020). *rpoC1* and *rpoC2* are two genes that encode for two subunits of the RNA polymerase (Bergsland and Haselkorn, 1991). Palenik and Haselkorn (1992) proposed the *rpoC1* as a valuable tool to clarify phylogenetic relationships among plant groups (Liston, 1992). Since that, it has been used together with other molecular markers to resolve phylogenetic relationships in many plant families (Downie et al., 1998; Messinger et al., 1999; Watson et al., 2000).

**FIGURE 2:**
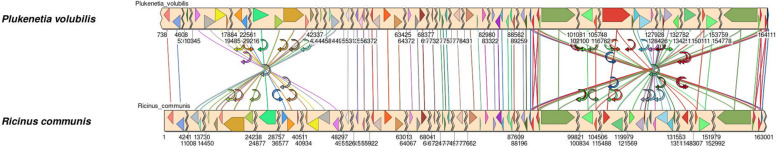
Synteny analysis between *Plukenetia volubilis* (top) and *Ricinus communis* (bottom) chloroplast genomes. Homolog genes are connected by arrows. Two inversions are observed on the LSC (left) and on the SSC (right). Jagged edges and accompanying base pair numbers denote the start and end of a contig region automatically collapsed by SimpleSynteny due to no genes being present to make the figure more compact.

Two large inversions were identified in the *P. volubilis* cpDNA when it was structurally compared with *R. communis* chloroplast (Figure 2). The first inversion is located in the middle of the LSC, spanning 39,426 bp, and flanking the genes *rps4* and *psbI*. A second more minor inversion of 17,493 bp changes the SSC orientation (relative to *R. communis*). This inversion affects the open reading frame of one of the *ycf1* copies and thus fragments it. This fragmented copy was annotated as a pseudogene. As no misassemblies were detected in the assembly (see section “Next-Generation Sequencing and Genome Assembly”), the observed rearrangements are from biological origin. Further assessment with Simple Synteny (Veltri et al., 2016) using the 60 longer genes showed how these inversions affected gene order mainly in the LSC (Figure 2). Both inversions are absent in the previously reported chloroplast (acc MF062253.1) because the sequence was generated with a guided assembly using *R. communis* chloroplast as a reference (Hu et al., 2018).

We performed a phylogenomic study with the chloroplast genomes available in the GenBank to the date for the family Euphorbiaceae members using ML reconstruction (Supplementary Figure 4). All of the Crotonoideae, Euphorbioideae, and Acalyphoideae subfamilies members were consistently grouped in the cladogram, and C1 was inner the tribe Plukenetieae next to *R. communis*. This was consistent with Hu et al. (2018), by suggesting that the general features of *P. volubilis* chloroplast genome compared to other phylogenetic relative chloroplasts have not been significantly different in terms of size or number of genes, but indeed have been significantly different in the structure of the genome presenting rearrangements in some populations of SI as it has been documented in other chloroplasts of the tribe Plukenetieae (Cardinal-McTeague and Gillespie, 2016).

### Phylogenetic Studies of Sacha Inchi Cultivars From Colombia

Alignments of *psbA-trnH^GUG^* IS sequences revealed nucleotide variation between SI Colombian cultivars (Table 1). The San Luis cultivar (C5) sequence showed multiple insertions between the nucleotides 321 and 345, while C1–C4 had a deletion in the same region (Table 1). Other studies have shown that *psbA-trnH^GUG^* IS could have potentially informative character (PIC) value relative to other cpDNA regions because it presumably shows considerable variability (>50%) between angiosperm lineages (Shaw et al., 2007). However, the marker resolution to unveil phylogenetics relationships has only been assessed at the tribe level (Plukenetieae in Cardinal-McTeague and Gillespie, 2016). At the genus level (*Plukenetia*), Cardinal-McTeague et al. (2019) supported two major groups (the pinnately- and palmately-veined clades) and five subclades within *Plukenetia* in perhaps the most profound phylogenetic time-dependent evolutionary framework study of the pantropical genus.

Authors mentioned above combined cpDNA (*matK* and *ndhF*) and nDNA (*KEA1* and *TEB*) markers to assess the phylogenetic relationships of *Plukenetia* spp. as well as its divergence in a geological time-scale. Our results in the *P. volubilis* cpDNA complete sequence showed that *matK* is a superimposed complex gene for the *trnK-UUU* and two antisense introns that flank the *trnK-UUU* as seen in other species such as *P. edulis* cpDNA (Cauz-Santos et al., 2017), and perhaps this nature of the gene is contributing to its resolution at this level. However, the single nucleotide variant (SNV) present in the Colombian cultivars and its associate PIC values across angiosperms lineages suggest that evolutionary changes might occur within the Colombian population *P. volubilis* due to domestication selection. Therefore, we hypothesize that the San Luis cultivar (C5) diverges from all other cultivars and that its evolutionary relationship with the other cultivars analyzed in this study could be revealed using the *psbA-trnH^GUG^* IS as a molecular marker.

**FIGURE 3:**
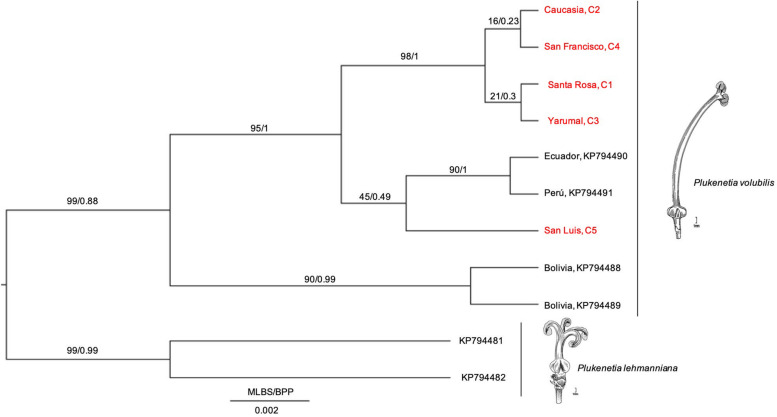
Phylogenetic hypothesis based on *psbA-trnH^GUG^* IS of the *P. volubilis* cultivars from Antioquia, Colombia, its relatives (KP794488-89 from Bolivia, KP794491 from Perú, and KP794490 from Ecuador, see Cardinal-McTeague and Gillespie, 2016) and selected outgroups (KP794481-82 representing *P. lehmanniana*). Phylogenetics methods were listed in Section “Materials and Methods.” Illustrations from Cathy Pasquale were adapted from Gillespie (1993).

To reveal intraspecific evolutionary relationships, we performed a phylogenetic analysis with an aligned matrix of 11 taxa and 362 bp (the five Colombian cultivars + four South American accessions + two Pterichis *lehmanniana* sequences as an outgroup). We inferred the ML and BI trees (Figure 3). Results showed that cultivars C1–C4 grouped in a unique monophyletic group, while San Luis Cultivar (C5) was found to diverge from the same ancestor as the Ecuador + Perú cultivars (Figure 3); however, this group (C5 + Perú + Ecuador) was not highly supported neither by MLB nor BPP values, indicating that their relationships could not be revealed in this study. Notwithstanding, a new supported clade (Colombia + Perú + Ecuador) suggests that cultivars diverged from an ancestor shared with Bolivian cultivars, suggesting that Colombian, Peruvian, and Ecuadorian cultivars might be sharing a common evolutionary history based on the *psbA-trnH^GUG^* IS marker.

According to this phylogenetic hypothesis, no differences among Colombian cultivars are observed (Figure 3). Notwithstanding, other relationships between Peruvian and Bolivian and Equatorial accessions of *P. volubilis* seem to display significant divergence. Furthermore, *P. lehmanniana*, as a sister group, is consistent with preceding studies (Cardinal-McTeague and Gillespie, 2016). Remarkably, the three main groups diverged with posterior probability values greater than 0.98 (Figure 3), which illustrates *psbA-trnH^GUG^* proper resolution for intraspecific relationships.

### Determination of the Organellar Inheritance Mode by Crossbreeding

Since cpDNA paternal contribution in angiosperms has not been reported extensively, and mitochondria are crucial during pollen tube germination (Twell et al., 2006), we assume that *P. volubilis* cpDNA is maternally inherited and its mtDNA is biparentally inherited. However, it is necessary to experimentally determine the cpDNA inheritance mode in *P. volubilis*, which is essential information for enabling successful genetic improvement programs, avoiding unwanted crosses with wild germplasm (Glick and Patten, 2017). It would appear suitable to develop genetically modified cultivars, harboring cpDNA modifications, avoiding gene scape. In cpDNA enhanced plants, higher expression of proteins is manifested, allowing them to overcome biotic and abiotic stress (Glick and Patten, 2017). In order to determine the cpDNA mode of transmission, *psbA-trnH^GUG^* IS was used as a chloroplast sequence marker in crossbreeding experiments of the present study’s cultivars. Therefore, solving cpDNA inheritance mode in *P. volubili*s is a stepping stone to decide how to develop modified cultivars eventually that could avoid unwanted crosses with wild germplasm (Glick and Patten, 2017).

As previously stated, *psbA-trnH^GUG^* IS sequences from Colombian cultivars indicated nucleotide variation (Table 1). Since then, the San Luis cultivar’s (C5) sequence has shown multiple insertions between 321 and 345 nucleotides, while C1–C4 have a deletion in the same region. This suggests the suitability of the cpDNA marker to discriminate among the local *P. volubilis* cultivars by the use of molecular techniques and particularly the cultivar C5 as the main parent in crossbreeding experiments. Many factors could be explaining the exhibited variability of *P. volubilis.* Additionally, the Peruvian Amazonia’s diverse ecological structure has allowed the domestication of native plants (such as *P. volubilis*) by keeping a high genetic variability during the last centuries (Rodríguez et al., 2010). In this context, the movement of non-genotyped seed and uncontrolled use of *P. volubilis* as a promising crop through different varied regions of South America might be possible, causing new varieties adapted to diverse habitat conditions (Correa et al., 1990; Gillespie, 1993). Besides, spotted SNVs (Table 1) can be tested for simultaneous occurrence in a *P. volubilis* interbreeding population and serve as additional statistical data supporting SNP discovery.

Sequences from the *psbA-trnH^GUG^* IS region in the F1 generation, derived from genetic crosses between the three parents (C1, C3, and C5), were obtained. Sequence analysis showed a maternal inheritance of the cpDNA since whenever the C5 was the maternal parental, the “. . . G . . . . . . T A T T T A . . . . . . T A” sequence was inherited to the F1 and the reciprocal crossing inherited the genotype C1 (Table 1), indicating a maternal inheritance mode of the cpDNA. This result is consistent with most angiosperm plants where cpDNA is uniparentally transmitted by the female parental, and thus it can be used to identify the maternal genome donor (Feitosa, 2017). Therefore, molecular understanding derived from cpDNA genome analysis suggests that the inheritance model *P. volubilis* is uniparental maternal, which supports cytogenetic analysis carried out in parallel.

### Determination of the Organellar Inheritance Mode by a Cytogenetic Approach

Around 80% of angiosperms display maternal inheritance mode of cpDNA, compared with the other 20% of angiosperms studied, which show a strong bent for plastid transmission from the male lineage, a phenomenon known as potential biparental plastid inheritance (PBPI); results mainly based on scanning plastid DNA in the male gametic cell with DAPI (Corriveau and Coleman, 1988; Zhang and Sodmergen, 2010). On the other hand, paternal inheritance in angiosperms has been observed in bare cases (Harris and Ingram, 1991; Testolin and Cipriani, 1997).

**FIGURE 4:**
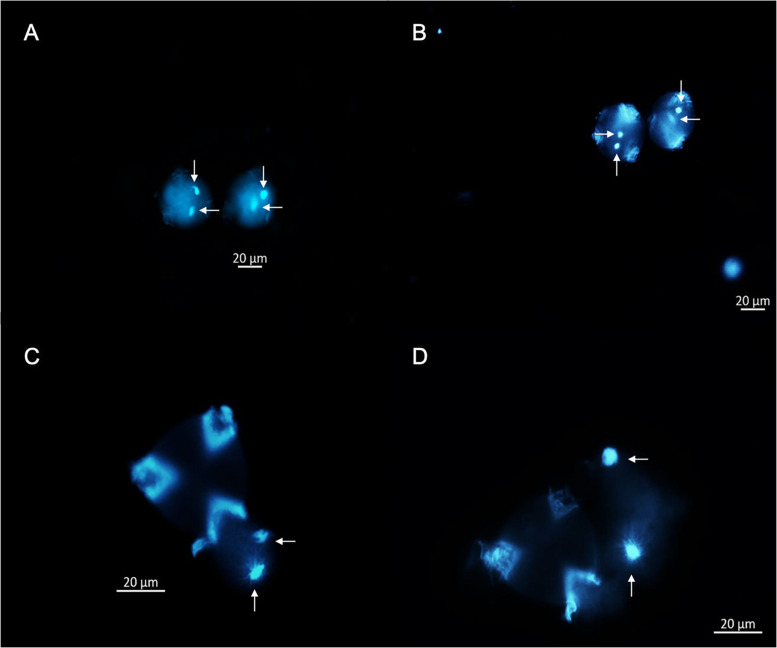
Germination stages of *P. volubilis* pollen grains using GM, stained using DAPI fluorochrome, and visualized under fluorescent microscopy. **(A)** (Stage 1), pollen grains not completely germinated, lacking pollen tube and stained directly. **(B)** (Stage 2), pollen grains showing very conspicuous germination apertures. **(C)** (Stage 3) pollen partially germinated with an observable migration of vegetative and germinative cells. **(D)** (Stage 4), pollen grains germinated and conspicuous pollen tubes with male germination unit (MGU) migrating across the tube. Germinative cells are illustrated in vertical arrows, and vegetative nuclei are pointed by the horizontal arrows. A and B were observed at 20X and C and D at 40X.

A cytological approach using DAPI staining was implemented in three steps to elucidate the oDNA inheritance mode of *P. volubilis*: (i) direct DAPI satin into germinated pollen grains; (ii) DAPI stain on alcohol fixed germinated pollen grains, and (iii) DAPI staining on histological slices of four flower bud’s developmental stages. During pollen grain germination, three aperture furrows’ appearance is conspicuous; these furrows expose feeble intine to the GM and are the proper places for tube germination (Figure 4A). Besides, observations of germinated pollen grains showed no evidence of plastid DNA or any oDNA granules; mtDNA is also included in any of the germination stages observed (Figure 4). Plant cytologists agree with the DAPI staining method as a fundamental approach, claiming that MGU migration through a pollinic tube could be considered the gametophyte’s final and active state (Twell et al., 2006). If plastids are absent throughout MGU migration, the zygote will be missing paternal plastids (Zhong et al., 2011). Therefore, adding molecular results, this study reports for the first time that *P. volubilis* oDNA is not paternally transmitted, making this scientific report one of the first profound advances in organellar inheritance for this species.

This experiment additionally shows that *P. volubilis* has a binucleate male gametic cell based on the diagnosis of pollen grains fixed with alcohol (Figures 4C,D). It exhibits a diffuse nucleus (vegetative) and a more defined one (germinative cell) at the end of gametogenesis. Notwithstanding, almost every plant species shows binucleated stereotypical pollen grains (70% of all plant spp.). According to Twell et al. (2006), trinucleate or binucleate pollen is a random feature among *Euphorbiaceae* (Brewbaker, 1967), therefore rendering a useful characteristic regarding pollen-based taxonomy.

Considering plastid DNA or oDNA granules, mtDNA is also included. Since DAPI staining is a non-selective DNA stained method, and no stained plastids were observed (Figure 4), it is suggested that *P. volubilis* mtDNA inheritance mode seems to be non-paternally as well. The mtDNA is independently trafficked from that of cpDNA. For instance, cpDNA and mtDNA of *Medicago sativa* are inherited biparentally and maternally, respectively (Forsthoefel et al., 1992), whereas those of *Musa acuminata* (banana) are inherited maternally and paternally, respectively (Fauré et al., 1994). These oDNA behaviors correlate well with the degradation or amplification of DNA in each organelle of the generative cell (Nagata et al., 1999); in the generative cells of *M. sativa*, cpDNA is amplified while mtDNA is degraded, whereas in *M. acuminata*, cpDNA is degraded while mtDNA is amplified (Twell et al., 2006).

**FIGURE 5:**
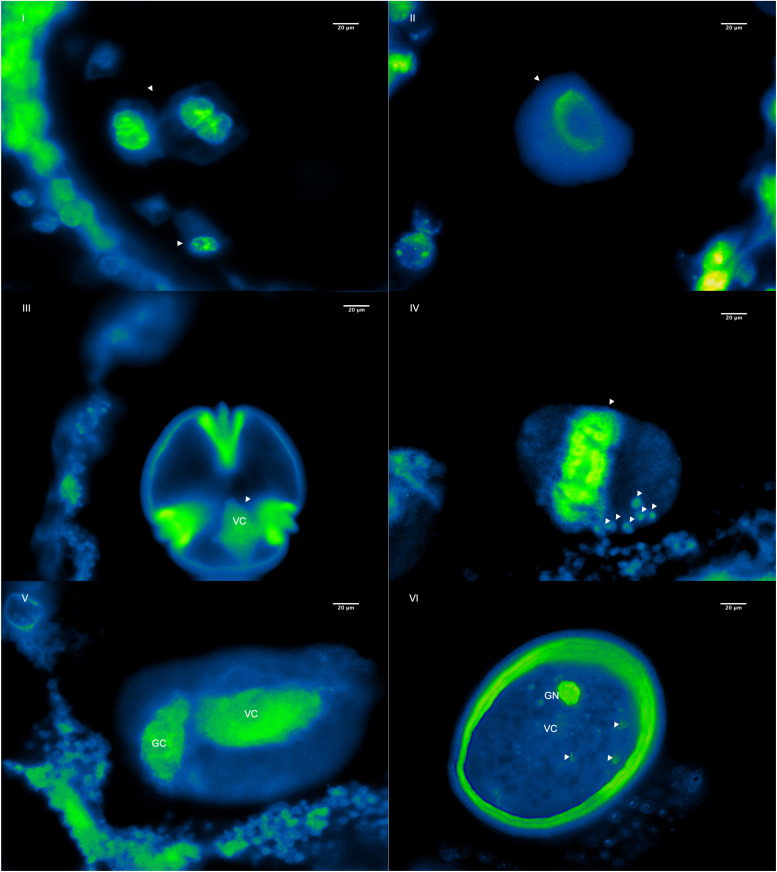
Six morphological stages of microsporogenesis and microgametogenesis are shown during the first four flower buds developmental stages of pollen cells of *P. volubilis* using fluorescent micrograph and DAPI stain. **(I)** First meiosis and formation of tetrads, **(II)** microspore release, **(III)** microspore during interphase, **(IV)** microspore undergoing the first pollen mitosis (PMI) and trafficked fluorescent oDNA granules (pointed out with the arrows), **(V)** formation of a generative cell (GC) and vegetative cell (VC), and **(VI)** mature pollen and cytoplasmic DNA decay and formation of the final MGU before anthesis. All observations are at 100X.

Histological slices of flower buds during four developmental stages were stained with DAPI to analyze the course of oDNA along pollen gametogenesis. Results showed oDNA decay during the switch of flower buds developmental stages III and IV (Figure 5). This decrease might be happening during pollen mitosis (PMI). Loss mechanisms are several, from restriction enzyme success to total cytoplasmic content suppression in a male gametophytic cell (Sager and Lane, 1972). Indeed, many cellular mechanisms controlling oDNA inheritance transmission have been proposed clearly, for instance: (1) physical exclusion of the organelle itself during PMI; (2) elimination of the organelle by the formation of enucleated cytoplasmic bodies (ECB); (3) autophagic degradation of organelles during male gametophyte development; (4) digestion of the organelle after fertilization; and (5) digestion of oDNA in generative cells just after PMI (Nagata, 2010). However, hypotheses (1) and (5) may encompass others and also explain better the maternally or non-paternally transmitted mechanism of oDNA (Nagata, 2010).

Since results showed an organelle decay in the very early stages of mitosis (Figures 5IV–VI), it is suggested that the oDNA loss mechanism in *P. volubilis* is primarily due to an organelle physical exclusion system. Indeed, many granular bodies are bordering outside pollen grains (Figure 5IV), suggesting an oDNA removal from pollen grains. Nonetheless, these observations do not, by themselves, definitively support this hypothesis. Additional evidence is needed, such as molecular studies showing how actin filaments or microtubules traffic organelle during microgametogenesis (Twell et al., 2006). Furthermore, loss mechanisms may not be mutually exclusive, so that multiple systems might be acting during pollen formation. It is reasonable to think that later, between the last stage of pollen formation (Figure 5VI) and pollen germination (Figures 4C,D), a second mechanism such as organelle digestion may be happening. Finally, histological slide observations should be taken carefully. They could eclipse natural phenomena because tridimensional bisected pollen grains may not accurately reflect what is inside.

## Conclusion

Supported by cpDNA genome and cytogenetic analysis, this study found that *P. volubilis* chloroplast’s inheritance model is uniparental maternal. With 164,111 bp length displaying two copies of an inverted repeat sequence (IRA and IRB), the *P. volubilis* cpDNA was reported completely *de novo* for the first time, filling a significant gap and need of studies for a species with great demand due to its high UFAs seeds content. When it was compared with *R. communis* cpDNA, two large inversions were identified in the *P. volubilis* cpDNA: the first inversion located in the middle of the LSC and the second one between the genes trnN-GUU. Furthermore, genomic analyses between *P. volubilis* cpDNA and other *Euphorbiaceae* cpDNA showed significant differences in the genome structure, including rearrangements. Here we reported a cpDNA *psbA-trnH^GUG^* IS as a molecular marker, which demonstrated the suitability to discriminate intraspecific relationships among regional *P. volubilis* cultivars, adding knowledge to understanding its genetic diversity. This outcome is crucial to track all the rudimentary SI ecotypes developed in tropical countries. Moreover, we consider that this work will contribute to generating stands for plant genetic improvement, primarily to further strategies based on chloroplast genetic transformation and understanding of evolutionary dynamics between organelle and nuclear genomes.

## Data Availability Statement

The datasets presented in this study can be found in online repositories. The names of the repository/repositories and accession number(s) can be found below: https://www.ncbi.nlm.nih.gov/genbank/ – MN912383, MN912384, MN912385, MN912386, and MN912387 (for markers), and MW591569 (for the chloroplast genome).

## Author Contributions

JÁ and DV-M were the principal investigators. SV-C contributed by extracting high molecular weight genomic DNA for ONT sequencing and analysis. CG-B and FG-C contributed equally to this work by collecting, processing, and analyzing pollen samples, and then observing, capturing, and processing images. CG-B, FG-C, and VR-R contributed by performing the DNA extractions from cultivars and the PCR experiments. SV-C performed all the bioinformatic analyses. JÁ, SV-C, DV-M, CG-B, and FG-C contributed to the manuscript’s writing. JÁ and VR-R guided every step of the experiments and helped with invaluable and profound suggestions. All authors contributed to the article and approved the submitted version.

## Conflict of Interest

The authors declare that the research was conducted in the absence of any commercial or financial relationships that could be construed as a potential conflict of interest.

## Abbreviations

AMSL: above mean sea level
cpDNA: chloroplast DNA
DAPI: 4’,6-diamidino-2-phenylindole
Δ AICc: Akaike information criterion
Δ BIC: delta of Bayesian information criterion
GC: generative cell
GM: germination media
IR: inverted repeat
IRA: inverted repeat A
IRB: inverted repeat B
IS: intergenic sequence
LSC: large single copy
MCMC: Markov chain Monte Carlo
MGU: male germination unit
mtDNA: mitochondrial DNA
oDNA: organellar DNA
ONT: Oxford Nanopore Technologies
PBPI: potential biparental plastid inheritance
PIC: potentially informative character
SNP: single nucleotide polymorphism
SNV: single nucleotide variant
SSC: small single copy
UFA: unsaturated fatty acid
UV: ultraviolet
VC: vegetative cell.
