# Supplementary material for: The Complete Chloroplast Genome of Plukenetia volubilis Provides Insights Into the Organelle Inheritance
Source: Front Plant Sci. 2021 Apr 23;12:667060. doi: 10.3389/fpls.2021.667060 (PMC8103035; doi:10.3389/fpls.2021.667060)
Supplement: Supplementary file 1 [file Data_Sheet_1.docx]

Supplemental methods

CpDNA *psbA-trnH^GUG^* marker amplification and sequencing

To amplify the cpDNA psbA-trnHGUG IS fragment from each cultivar of *P. volubilis*, *trnH^GUG^* (5’-CGCGCATGGTGGATTCACAATCC-3’) (Tate and Simpson, 2003) and *psbA* (3’-GTTATGCATGAACGTAATGCTC-5’) (Sang et al., 1997) primers were synthetized and then used. Amplification reagents concentrations for 25 µL of reaction were: Buffer taq 1X, MgCl_2_ 2.3 mM, dNTPs 0.4 mM, primers 0.3 µM, DNA 10-100 ng, Taq polymerase 1.2 U adapted from Cardinal-McTeague and Gillespie (2016). The following PCR Touchdown profile was run in C1000 Touch thermal cycler (Bio-Rad, CA, USA): initial denaturalizing at 94°C for 3 min, 24 cycles of (i) denaturalizing at 94°C for 45 s (ii) annealing at 60.5°C for 45 s and (iii) extension at 72°C for 1 min, follow by 10 cycles of: (i) denaturalizing at 94°C for 45 s (ii) annealing at 59.5°C for 45 s and (iii) extension at 72°C for 1 min, finally an extension at 72 ºC for 10 min. PCR products were checked in a 1 % agarose gel and stored at -20 ºC until sequencing.

DNA extraction for sequencing

Plant DNA extraction was performed following a modification of [Paper de tesia]. Lysis buffer from Schalamun and Schwessinger (Schalamun and Schwessinger, 2017) was also used. Gravities and time were modified in the centrifugation steps, to decrease the mechanical shearing stress over the DNA, ensuring an unchanged decanting time.

Around 150 mg of freshly harvested leaves were ground in liquid nitrogen with a mortar and pestle and immediately transferred to 1 ml, previously heated to 64°C, of either CTAB (100 mM Tris-HCl pH 8.0, 1.4 M NaCl, 20 mM EDTA, 2x CTAB, 0.1% (v/v), β-mercaptoethanol) or lysis (2% PVP40, 500 mM NaCl, 100 mM TRIS-HCl pH 8, 50 mM EDTA, 1.25% SDS, 1%(w/v) sodium metabisulfite, 5 mM Dithiothreitol (DTT)) buffer, and incubated at 37 °C for 30 min. The extract was added with 1μlof proteinase K (10mg/ml) and incubated another 30 min at the same temperature. After a 5 min cooldown, 0.3 volumes of 5M potassium acetate were added and the extract was centrifuged (8000 g for 12 min at 4 °C). The supernatant was transferred to afresh tube, 1 volume of chloroform-isoamyl alcohol (24:1) was added and gently mixed by inversion before centrifuging (8000g for 12 min at 4 °C). The previous step was performed twice. The aqueous phase was transferred to a fresh tube, 0.1 volumes of 3M sodium acetate were added and the tube was gently mixed by inversion before 1 equivalent volume of isopropanol was added. The extract was incubated at -20 °C overnight and then centrifuged (10000g for 10 min at 4 °C). The resulting DNA pellet was washed with 200 μl of 70% ethanol and centrifuged (10000g for 10 min at 4 °C) twice before left to dry at room temperature for 30 min. The pellet was resuspended in 40 μl of nuclease-free water and 1μlof RNAse A (1 mg/ml) was added before a final incubation at 37 °C for 1 hour.

Extract quality was evaluated using gel electrophoresis for size estimation, spectrophotometry (A260/A280 and A260/A230 ratios) for purity estimation, and Qubit for total DNA extracted. DNA samples with a A260/A280 ratio close to 2 and a A260/A230 ratio above 1.5 were kept.
