# Supplementary material for: The Complete Chloroplast Genome of Plukenetia volubilis Provides Insights Into the Organelle Inheritance
Source: Front Plant Sci. 2021 Apr 23;12:667060. doi: 10.3389/fpls.2021.667060 (PMC8103035; doi:10.3389/fpls.2021.667060)
Supplement: Supplementary file 2 [file Data_Sheet_2.docx]

Supplementary material

**Supplementary Figure S1** Geographical sample points of Sacha inchi cultivars farmlands across Antioquia, Colombia. Labels 1-5 correspond to the farmland town closer to the collection site of the cultivars (C1-C5). C1: Santa Rosa de osos, 6° 35' 42,9" N, 75° 13' 49,6" W; C2: Caucasia, 7° 58' 58,9" N, 75° 10' 4,1" W; C3: Yarumal, 7° 2' 31,3" N, 75° 21' 5,6" W ; C4: San Francisco, 5° 55' 14,2" N, 74° 52' 5,8" W; C5: San Luis, 6° 1' 30,7" N, 74° 58' 6,1" W. Google Earth ©.


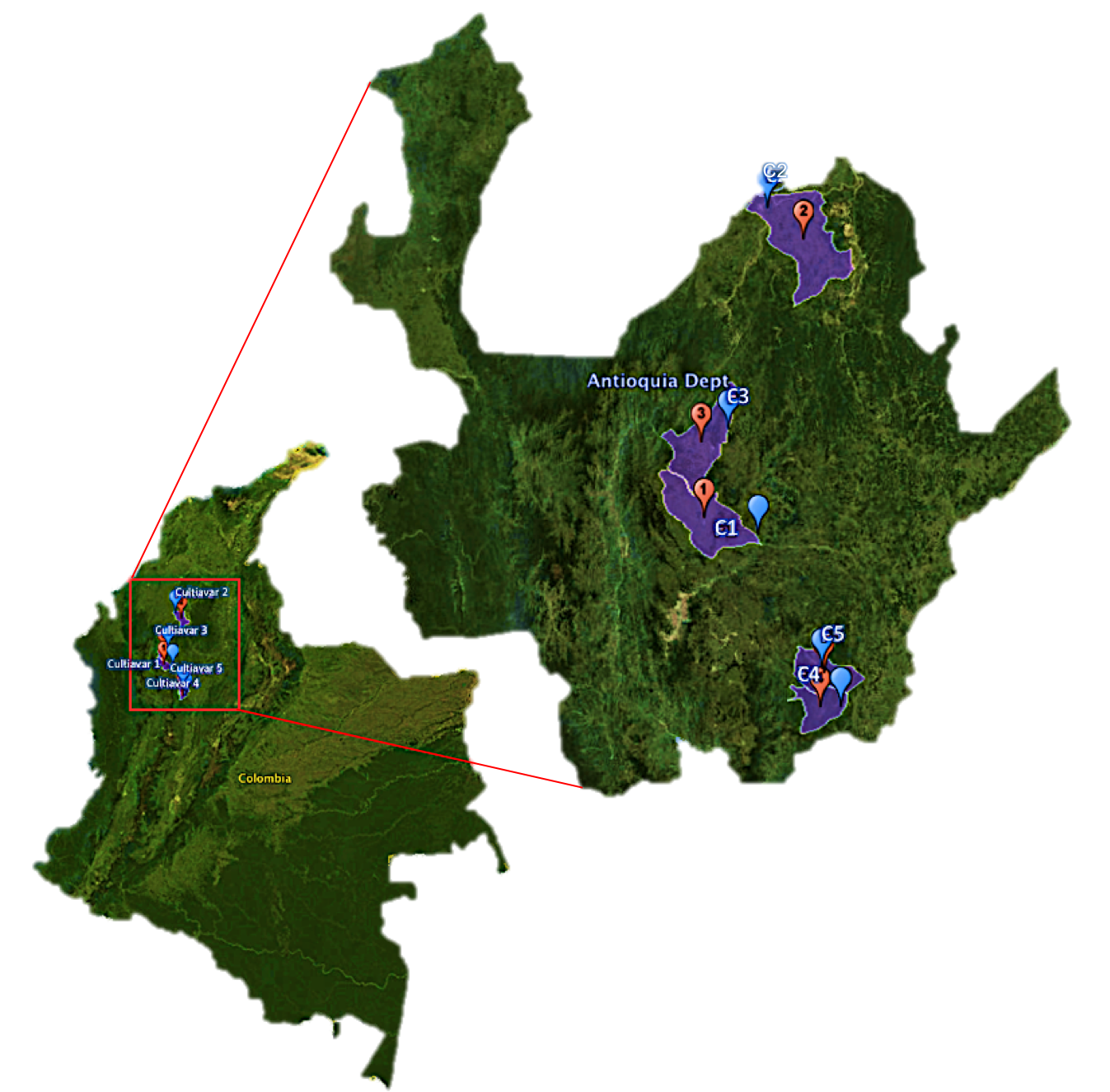


**Supplementary Table S1.** Cultivars of *P. volubilis* sampling points and accession numbers of *psbA-trnHGUG*IS marker in NCBI database. Altitudes range from 685 to 1501 MASL and habitat vary among all cultivars. Both leaf tissue and seeds were collected.

| **Cultivar ID (Isolate)** | **Country:Location, State** | **Altitude (MASL)** | **Coordinates** | **Accession number (NCBI)** |
| --- | --- | --- | --- | --- |
| C1 | Colombia:Santa Rosa, Antioquia | 1501 m | 6° 35' 42,9" N, 75° 13' 49,6" W | MN912383 |
| C2 | Colombia: Caucasia, Antioquia | 44 m | 7° 58' 58,9" N, 75° 10' 4,1" W | MN912384 |
| C3 | Colombia: Yarumal, Antioquia | 1118 m | 7° 2' 31,3" N, 75° 21' 5,6" W | MN912385 |
| C4 | Colombia: San Francisco, Antioquia | 561 m | 5° 55' 14,2" N, 74° 52' 5,8" W | MN912386 |
| C5 | Colombia:San luis, Antioquia | 685 m | 6° 1' 30,7" N, 74° 58' 6,1" W | MN912387 |

**Supplementary Figure S2.** Coverage of reads of ONT mapped in the cpDNA assembled of *Plukenetia volubilis*. A continuous smooth coverage of an average 3917x depth was found after run minimap2 with 956508 long reads.

LSC IRA SSC IRB

**
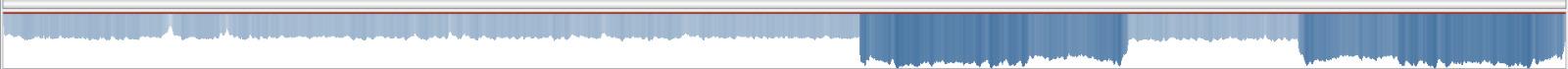
**

**Supplementary Table S2.** Comparison between closely cpDNAs reported in the Genbank.

|  | P.volubilis (MW591569) | P. volubilis (MF062253.1) | R. communis (NC_016736.1) |
| --- | --- | --- | --- |
| Size (bp) | 164,111 | 161,733 | 163,161 |
| Number of genes | 131 | 130 (122 in original .gbk) | 131 |
| GC content (%) | 35,79 | 36,23 | 35,74 |
| CDS(%genome) | 86 (47,89) | 86 (49,58) | 86 (48.72) |
| tRNAs | 36 | 36 | 37 |
| rRNAs | 4x2 | 4x2 | 4x2 |
| Introns(%genome) | 20(9,10) | 18(8,38) | NR |

**Supplementary Table S3.** Gene content of the *Plukenetia volubilis* chloroplast genome according to respective categories.

| **Category** | **Gene** | **#Unique** |
| --- | --- | --- |
| Subunits of photosystem I | *psaA, psaB, psaC, psaI, psaJ* | 5 |
| Subunits of photosystem II | *psbA, psbB, psbC, psbD, psbE, psbF, psbH, psbI, psbJ, psbK, psbL, psbM, psbT, psbZ* | 14 |
| Subunits of cytochrome b/f complex | *petA, petB, petD, petG, petL, petN* | 6 |
| Subunits of ATP synthase | *atpA, atpB, atpE, atpF, atpH, atpI* | 6 |
| Large subunit of rubisco | *rbcL* | 1 |
| Subunits of NADH-dehydrogenase | *ndhA, ndhB, ndhC, ndhD, ndhE, ndhF, ndhG, ndhH, ndhI, ndhJ, ndhK* | 11 |
| Proteins of large ribosomal subunit | *rpl2, rpl14, rpl16, rpl20, rpl22, rpl23, rpl32, rpl33, rpl36* | 9 |
| Proteins of small ribosomal subunit | *rps2, rps3, rps4, rps7, rps8, rps11, rps12, rps14, rps15, rps16, rps18, rps19* | 12 |
| Subunits of RNA polymerase | *rpoA, rpoB, rpoC1, rpoC2* | 4 |
| Cytochrome c biogenesis | *ccsA* | 1 |
| Maturase | *matK* | 1 |
| Protease | *clpP* | 1 |
| Envelope membrane protein | *cemA* | 1 |
| Conserved hypothetical genes | *ycf1,ycf2, ycf3, ycf4* | 4 |
| Ribosomal RNAs | *rrn4.5, rrn5, rrn16, rrn23* | 4 |
| Tranfer RNAs | *see report of trnascan-se* | 36 |
| Fragment genes | ycf1 | 1 |
| *Duplicated in annotation | ndhB, ndhG, rpl2, rpl23, rps12, rps19, rps7, rrn16, rrn23, rrn4.5, rrn5, trnA-UGC, trnH-GUG, trnI-GAU, trnL-CAA, trnM-CAU, trnN-GUU, trnR-ACG, trnV-GAC, ycf1, ycf2 |  |
| total single-copy genes |  | 87 |

**Supplementary Table S4.** Evolutionary substitution model evaluation.

| Criterium | Model | Score | Weight |
| --- | --- | --- | --- |
| BIC | F81+G4 | 1944.9471 | 0.4263 |
| AIC | TVM+I+G4 | 18094.4477 | 0.1264 |
| AICc | TVM+I+G4 | 1813.447 | 0.0868 |

**Supplementary Figure S3.** Chromosome comparison between Sacha inchi cpDNA and *Ricinus communis* cpDNA using nucmer algorithm.


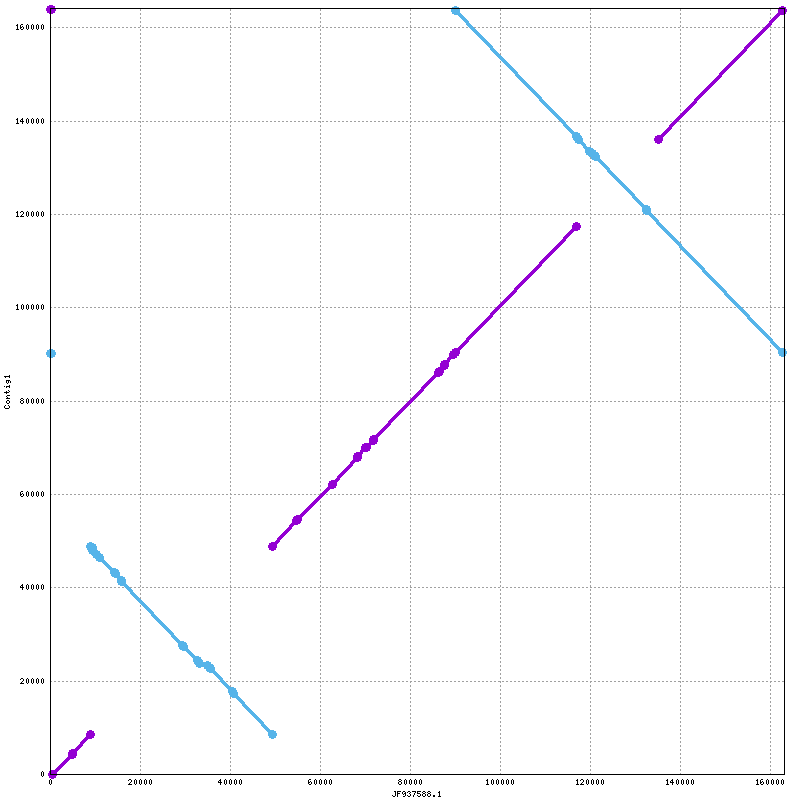


**Supplementary Figure S4**

Phylogenomic analysis of the *Euphorbiaceae* family. Alignments outputted by GeSeq (Tilich et al., 2017) were concatenated with homemade python scripts, then a phylogenetic Maximum likelihood analysis was performed in RAxML, based on GTRCAT model. To estimate the level of support for the ML topology, bootstrap was performed on 100000 replicates. Generated trees were visualized in Figtree. Taxa used for the analysis were: NC034803 - *Vernicia fordii,* NC041102 - *Deutzianthus tonkinensis,* NC040113 *- Croton tiglium,* NC012224 *- Jatropha curcas,* NC015308 - *Hevea brasiliensis,* NC010433 - *Manihot esculenta,* NC033910 *- Euphorbia esula,* NC042193 *- Euphorbia tirucalli,* MF062253 *- Plukenetia volubilis,* NC016736 *- Ricinus communis*

**
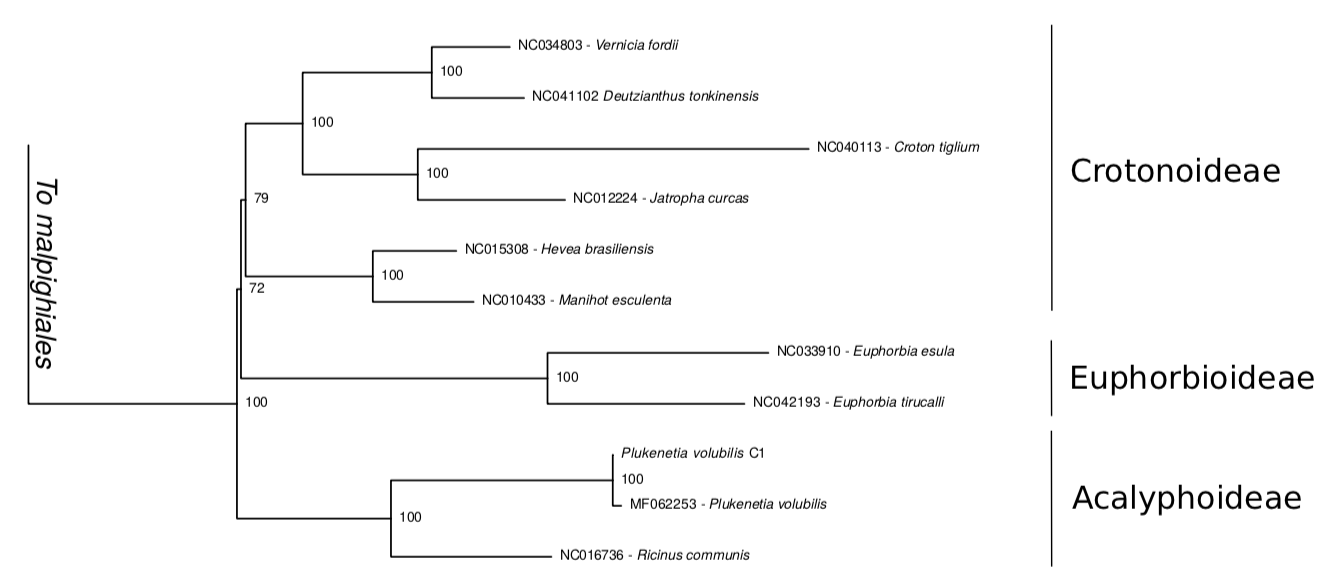
**

Tillich M, Lehwark P, Pellizzer T, Ulbricht-Jones ES, Fischer A, Bock R and Greiner S (2017) GeSeq – versatile and accurate annotation of organelle genomes. [Nucleic Acids Research](https://academic.oup.com/nar/article/45/W1/W6/3806659/GeSeq-versatile-and-accurate-annotation-of) [45: W6-W11](https://academic.oup.com/nar/article/45/W1/W6/3806659/GeSeq-versatile-and-accurate-annotation-of)
